# On Making Experiments on Living Animals

**Published:** 1817-02

**Authors:** 


*For the London Medical and Physical Journal.*

### *On making Experiments on Living Animals;* by E. H.

IN No. 211 of your Journal, I offered a few suggestions on the impropriety of making wanton and unnecessary experiments on living animals. In the Journal for December 1816, p. 457, a correspondent has stepped forward on the other side the question, and censures your having agitated it at at all. The subject which has called forth your animadversions is not of a trifling and uninteresting nature, but involves in it considerations of the first importance to the cause of humanity. The candid expression of your disapprobation is said to have excited a smile at your expence; but your sentiments will not be so easily controverted; and, whatever impression they may make on those who have a predilection for the custom, your conduct in this instance has certainly been influenced by a spirit far more philosophical than that which has directed the majority of the experiments on animals. If you had the merit of leading the way, you are by no means singular in your opinion, as two respectable recent publications* amply testify, in which, much to the credit of their respective authors, the practice is decidedly deprecated.

Notwithstanding the subject has been "so often introduced," as it possesses considerable interest, and as it has not yet received that ample investigation which might supersede the necessity of further discussion, I must beg leave to lay before your readers a few additional remarks.

In taking a view of the manner in which this practice can tend to benefit medical science, it must either be, first, in ascertaining the qualities and effects of remedies; or, secondly, in improving the practice and principles of surgery; or, thirdly, in the extension of physiological knowledge.

With respect to the first object, it will be evident, at first sight, that medicine can derive little aid from this source. The only department where it is applicable is, in discovering the effects of, and the antidote for, the different classes of poisons; and of its use here, the indefatigable Orfila has sufficiently availed himself. His laborious and persevering exertions have certainly placed him at the head of the writers on Toxicology; but the judgment of one of his critics† is not too severe, when he observes, that the information to be derived from the results of his experiments is not equal to the sufferings inflicted on the unfortunate animals that were the subjects of them. In surgery, a few facts have been discovered illustrative of the powers of nature in overcoming injuries in some of the most important parts of the system, such as the arteries, intestines, &c. But which of the operations on the human body has received any improvement from, or in what disease has a more judicious plan of treatment been suggested by these multifarious experiments?

---

* Vide Carson's Inquiry into the Causes of the Motion of the Blood; and Male's Epitome of Forensic Medicine.

periments? Physiology has undoubtedly been more indebted to this method of investigation than any other branch of medicine, and has been enriched by discoveries that would not have been brought to light by any other means. We are hence made acquainted with the phenomena of the nervous system, of the generation of animal heat, and of the process of digestion; the explanation and elucidation of which has formed a splendid æra in medical literature.

To the remark, that "it is possible to be humane without sacrificing the interests of science," we cordially acquiesce. This is the position we endeavour to maintain; we are not insensible to the attractions of science, or indifferent to its advancement. It is not the use, but the abuse of it, that is to be condemned. It is not wished to prohibit any experiments tending to practical utility; and, surely, Amicus will admit, that the dictates of humanity are to be preferred to the gratification of an unprofitable curiosity. Man is emphatically termed the Lord of the Creation, and, while he ought to feel grateful for his elevation over all other animals, he can claim no authority for causing them unnecessary anguish. Let those, therefore, who are disposed to sanction such proceedings reflect on the probable result of their pursuits; and not imagine that they are labouring in the cause of science, when they are amusing themselves with hypothetical and useless speculations, or merely incited by the vanity of recording them.

---

### *On the Minim Measure.*

The paper by Mr. Ward, in your Journal for November, excited my surprise, and undoubtedly that of many other of your readers. If Mr. W. would take the trouble of consulting the Pharmacopœia, he will perceive, that the reasons he urges against the use of the minim measure, are the same that induced the College of Physicians judiciously to adopt it: viz. the variation and uncertainty of drops. The different quantity of the various pharmaceutical preparations contained in the same number of drops, is well known to every one at all conversant with the dispensing of medicine. Should any prescribe the minim to be used with the notion of its being intended as a synonyme for drop, their error is referrable to their own carelessness and ignorance, and not to a defect in the principle of the measure. Besides the reference arising from the nature of the article, the quantity will be influenced by other circumstances, as the size of the bottle, the quantity contained in it, and the formation of its lip. It is also to be feared, that the same nicety and exactness is not always manifested in compounding, that

Mr. W. would exercise in making his critical experiments. From this uncertainty in the dose, arising from the custom of dropping medicines, the minim measure has been hailed by many, with great satisfaction, as a valuable acquisition to the pharmaceutist. The assertion, that it can be only condered as an addition to the number of the apothecary's utensils, is the more extraordinary coming from the pen of so intelligent a practitioner as Mr. Ward.

*Bishopsgate-street; Dec.* 26, 1816.

